# Occurrence of Fungicides in Vineyard and the Surrounding Environment

**DOI:** 10.3390/molecules26206152

**Published:** 2021-10-12

**Authors:** Meruyert Sergazina, Lua Vazquez, Maria Llompart, Thierry Dagnac

**Affiliations:** 1 CRETUS, Department of Analytical Chemistry, Nutrition and Food Science, University of Santiago de Compostela, E-15782 Santiago de Compostela, Spain; sergazina.meruyert@gmail.com (M.S.); lua.vazquez.ferreiro@usc.es (L.V.); 2 Department of Chemistry, Institute of Natural Science and Geography, Abai Kazakh National Pedagogical University, Almaty 050010, Kazakhstan; 3 Galician Agency for Food Quality—Agronomic Research Centre (AGACAL-CIAM), Unit of Organic Contaminants, Apartado 10, E-15080 A Coruña, Spain

**Keywords:** fungicides, GC-MS/MS, vineyard, grapes, leaves, environmental matrices, ultrasound-assisted extraction

## Abstract

Seventeen fungicides were determined in different matrices from vineyard areas, including vine leaves, soils, grapes and water, using gas chromatography coupled to tandem mass spectrometry (GC-MS/MS). For leaf analysis, ultrasound-assisted extraction (UAE) was performed evaluating different solvents. UAE was compared with other extraction techniques such as vortex extraction (VE) and matrix solid-phase dispersion (MSPD). The performance of the UAE method was demonstrated on vine leaf samples and on other types of samples such as tea leaves, underlining its general suitability for leaf crops. As regards other matrices, soils were analyzed by UAE and microwave-assisted extraction (MAE), grapes by UAE and waters by SPE using cork as the sorbent. The proposed method was applied to 17 grape leaf samples in which 14 of the target fungicides were detected at concentrations up to 1000 μg g^−1^. Furthermore, the diffusion and transport of fungicides was demonstrated not only in crops but also in environmental matrices.

## 1. Introduction

According to the European Commission, pesticides are substances that are employed to prevent, destroy and control a harmful organism or infection, or protect plants or plant products during production, storage and transportation. Plant protection products (PPPs) are pesticides specifically applied for the protection of crops or desirable or valuable plants [1]. Fungicides are a type of pesticide (PPP) used to inhibit fungal growth, which can seriously damage crops and plantations of different types [2]. Although their use is indispensable for food safety, concerns have been raised about their presence in different consumption products and in the surrounding environment (water, air and soil) where they are employed [3,4,5,6,7]. This can represent a health hazard to consumers and a risk for the surrounding environment [8,9].

Viticulture is an industry where fungicides are widely employed to prevent fungal infections associated with Vitis plants. The main infections treated are grey rot (*Botrytis cinerea*), downy mildew (*Plasmopara viticola)* and powdery mildew (*Uncinula necator*) [10]. These diseases proliferate more in places with a warm and humid climate, such as Galicia (Northwest Spain). Currently, in order to protect both the environment and human health from adverse effects, there are limitations on the presence of pesticides in groundwater [11] and in surface water, for which Environmental Quality Standards (EQS) set concentration thresholds of several contaminants, including pesticides.

In addition to the above-mentioned possible dissemination into the environment (water and soil), it is important to identify and quantify fungicide residues in leaves and grapes of the vine crops on which this class of pesticides is sprayed. The fungicide concentration thresholds must comply with maximum residue levels (MRLs) that are established in food and feed of plant and animal origin [12], and especially in vine leaves as well as in vine and table grapes. In order to obtain the current MRL for any active substance used in plant protection products, it is advised to access the EU Pesticides Database, which allows users to search for any updated related information [13]. It is worth noting that MRLs for most fungicides in table and vine grapes are surprisingly high, many of them being at the mg kg^−1^ level or higher, while most pesticide MRLs in other foodstuffs are generally between 0.01 and 0.05 mg kg^−1^.

QuEChERS (a quick, easy, cheap, effective, rugged and safe method) is the preferred extraction technique for the determination of fungicides in vine leaves [14,15]. Besides, for other leaf samples, such as tea, other methodologies have also been employed for the extraction of pesticides, including solid-phase microextraction (SPME), solid-phase extraction (SPE), matrix solid-phase dispersion (MSPD), dispersive solid-phase extraction (d-SPE), pressurized liquid extraction (PLE) and liquid-liquid extraction (LLE) [16,17,18,19]. However, for the isolation of these compounds in grape samples, QuEChERS, solid liquid extraction (SLE), MSPD, SPE, dSPE, PLE, LLE, dispersive LLE (dLLE), ultrasound-assisted extraction (UAE) and microwave-assisted extraction (MAE), among others, have been used [20,21] In addition, UAE, PLE, MAE and MSPD were applied for the extraction of fungicides in soil samples [22,23,24]. Regarding water samples, different approaches like SPE, SPME or fabric phase sorptive extraction (FPSE) have been employed [2,8,25].

The most commonly used analytical techniques for fungicide analysis are liquid chromatography (LC) and gas chromatography (GC). The hybridization of these two separation techniques with mass spectrometry allows not only a good separation of these compounds, but also their excellent identification and accurate quantification [25,26,27].

The main goal of this study was the development and validation of a UAE method for the determination of 17 fungicides in vine leaf samples. Furthermore, several analytical approaches were applied in different matrices of vineyard and the surrounding environment (grapes, soil and water) for the determination of the same target compounds. This is the first study dealing with the occurrence assessment of the target fungicides in the four different matrices covered. Gas chromatography coupled to tandem mass spectrometry was employed to separate, identify and quantify the seventeen fungicides.

## 2. Materials and Methods

### 2.1. Chemicals and Materials

The 17 target fungicides, their CAS numbers, retention times and MS/MS transitions are summarized in Table S1. Acetonitrile, ethyl acetate, methanol and ultrapure water MS grade were provided by Scharlab (Barcelona, Spain), acetone and hexane by Sigma-Aldrich Chemie (Stenheim, Germany). Florisil (mesh size: 60–100 μm) and glass wool were purchased from Supelco (Stenheim, Germany) and sand (mesh size 200–300 μm) by Scharlab. Individual stock solutions of each fungicide were prepared in methanol. Further dilutions and mixtures were prepared in ethyl acetate or acetone. All solutions were stored in amber glass vials and protected from light at −20 °C. All solvents and reagents were of analytical grade.

### 2.2. Samples

Vine leaves, grapes, soil and water samples were collected (summer 2019) in three vineyard areas from Galicia (NW Spain) and were treated with fungicides about two weeks before, except those samples coming from the experimental study employing a drone for fungicide application.

About 20 g of leaves, 50 g of grapes and 100 g of soil below the vine plant were collected. The three wine grape varieties were Treixadura, Albariño and Loureiro.

For water samples, approximately 200 mL was collected in containers that were placed under the crops during rainy days and when they were irrigated.

Leaves and grapes were stored at −20 °C and soils and water at 4 °C, all of them protected from light until their analysis. Tea samples were purchased from local markets in Kazakhstan and stored at room temperature in the dark until their analysis.

### 2.3. Experimental Procedure

For UAE (50 kHz, 25 °C), 0.2 g of sample (leaves and grapes) was placed in a 10 mL vial and 2 mL of ethyl acetate was added. In the case of leaves for VE, the same conditions as UAE were applied. After that, the extraction was performed for 10 min. Regarding the MSPD procedure, 0.2 g of leaf samples were blended with 0.8 g of dispersing agent (florisil or sand) and transferred into a 2 mL polypropylene syringe with glass wool in the bottom. The column was eluted by gravity with 2 mL of EtAc. All extracts were filtered through 0.22 μm PTFE filters and diluted 4 times before GC–MS/MS analysis.

Soil samples were also analyzed by UAE and MAE. For MAE, 0.8 g of soil and 8 mL of ACN were placed in the vessels. Recommended extraction conditions from USEPA microwave extraction method 3546 were applied [28]. Since the UAE method allowed the use of a lower amount of sample, it was performed applying the same conditions as those used for grapes and leaf samples: 0.2 g of soil with 2 mL of EtAc in a 10 mL vial were placed in the ultrasound bath at 50 kHz and 25 °C for 10 min. All extracts were filtered (0.22 μm PTFE filters) and diluted (4 times) prior to analysis.

As regards the analysis of the water samples, a previously developed method based on SPE was carried out [8]. For that, 50 mg of re-granulated cork powder was packed into a 2 mL polypropylene cartridge containing a cellulose filter at the bottom. Under vacuum conditions (2 mL min^−1^), employing an SPE manifold (VisiprepTM, Supelco, Bellefonte, PA, USA), the sorbent was conditioned by washing with 2 mL of MeOH followed by 2 mL of ultrapure water, and then 20 mL of water sample was loaded through the cartridge. Afterwards, SPE cartridges were air dried for 15 min under vacuum to remove the residual water. Finally, the elution was carried out by gravity collecting 1 mL of EtAc in a volumetric flask, transferred to a 1.8 mL vial and directly analyzed by GC-MS/MS.

### 2.4. GC-MS/MS

Analyses were carried out employing a Thermo Scientific Trace 1310 gas chromatograph coupled to a triple quadrupole mass spectrometer (TSQ 8000) with an autosampler IL 1310 from Thermo Scientific (San Jose, CA, USA). Separation was performed on a Zebron ZB-Semivolatiles (30 m × 0.25 mm i.d. × 0.25 μm film thickness) supplied by Phenomenex (Torrance, CA, USA). Helium (purity 99.999%) was employed as a carrier gas at a constant flow of 1 mL min^−1^. The GC oven temperature was programmed from 60 °C (held 1 min), to 220 °C at 20 °C min^−1^, to 260 °C at 10 °C min^−1^ (held 3 min) and finally to 290 °C at 20 °C min^−1^ (held 10 min). The total run-time was 27.5 min. Pulsed splitless mode (200 kPa, held 1.2 min) was employed for injection. The injector temperature was set at 270 °C, and the injection volume was 1 μL. The mass spectrometer detector (MSD) was operated in the electron ionization (EI) positive mode (+70 eV). The temperatures of the transfer line and the ion source were set at 290 °C and 350 °C, respectively. The filament was set at 25 μA and the multiplier voltage was 1460 V. Selected reaction monitoring (SRM) acquisition mode was implemented monitoring 2 or 3 transitions per compound (see Table S1) for an unequivocal identification and quantification of the target fungicides. The system was operated by Xcalibur 2.2, and Trace Finder™ 3.2 software (Thermo Scientific).

### 2.5. Statistical Analysis

Basic and descriptive statistical analyses were performed using the software package Statgraphics Centurion XVII (Manugistics, Rockville, MD, USA).

## 3. Results and Discussion

### 3.1. Chromatographic Analysis

The instrumental parameters were adapted from Celeiro et al. (2020) [8]. Extracts were analyzed by GC-MS/MS. The applied instrumental parameters can be found in Section 2.4 and see also MS/MS transitions in Table S1.

The method was evaluated in terms of linearity, linear range, instrumental detection limits (IDLs) and instrumental quantification limits (IQLs), and inter-day and intra-day precision. The results are shown in Table 1 demonstrating suitable performance.

Calibration curves were obtained using standard solutions prepared in ethyl acetate (EtAc) containing the 17 target fungicides at different levels, covering a concentration range from 0.02 to 1000 μg L^−1^ with 15 levels and 3 replicates per level (see specific range for each fungicide in Table 1). The method showed a good linearity with coefficients of determination (R^2^) higher than 0.9937 for all compounds.

The GC-MS/MS precision was evaluated within a day (*n* = 4) and amongst days (*n* = 6) at different concentration levels. Relative standard deviation (RSD) values for 10 μg L^−1^ are given in Table 1, and they were in general lower than 8%. IDLs and IQLs were calculated as the compound concentration giving a signal-to-noise ratio S/N = 3 and S/N = 10, respectively, and they were at the sub ng mL^−1^ for most compounds.

The chromatographic response was compared using different solvents including acetonitrile (ACN), methanol (MeOH), EtAc and Hexane:Acetone (50:50, *v/v*). Figure S1 shows the chromatographic area counts for the 4 solvents (100 μg L^−1^). Similar responses were achieved for all compounds, excluding tolylfluanid and folpet whose responses were lower and even not detected (folpet) when ACN was used.

### 3.2. Extraction of Vine Leaves

**Figure 1:**
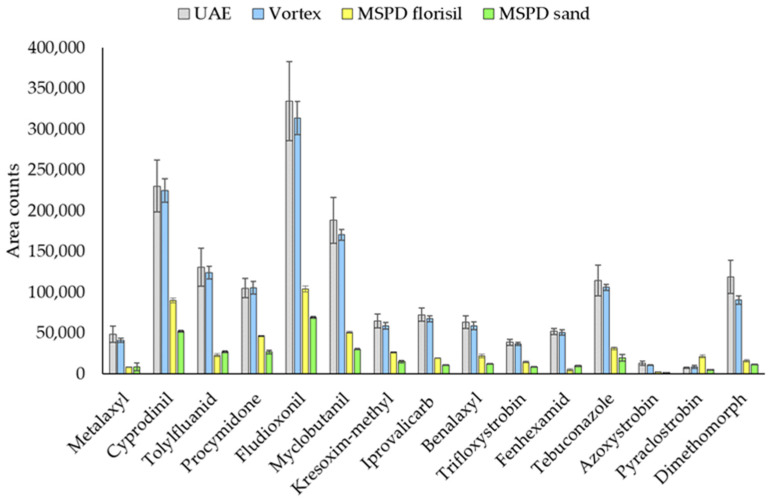
Fungicide responses for UAE, VE and MSPD using florisil or sand as dispersing agents.

Three miniaturized, fast and simple extraction techniques, which allow the use of a small amount of sample and solvent, were compared for the extraction of vine leaves: ultrasound-assisted extraction (UAE), vortex-assisted extraction (VE) and matrix solid-phase dispersion (MSPD). The procedures carried out for the different techniques are described in Section 2.3. Responses for each extraction procedure are represented in Figure 1.

As can be seen, responses up to 3 times lower were obtained with MSPD than with the other techniques for all target compounds, regardless of the dispersing agent used. However, UAE and VE extraction offered quite similar high responses for all target compounds. For this reason, MSPD was discarded as an extraction technique for the analysis of fungicides in vine leaves and UAE and VE were selected for further experiments.

#### 3.2.1. Extraction Solvent

**Figure 2:**
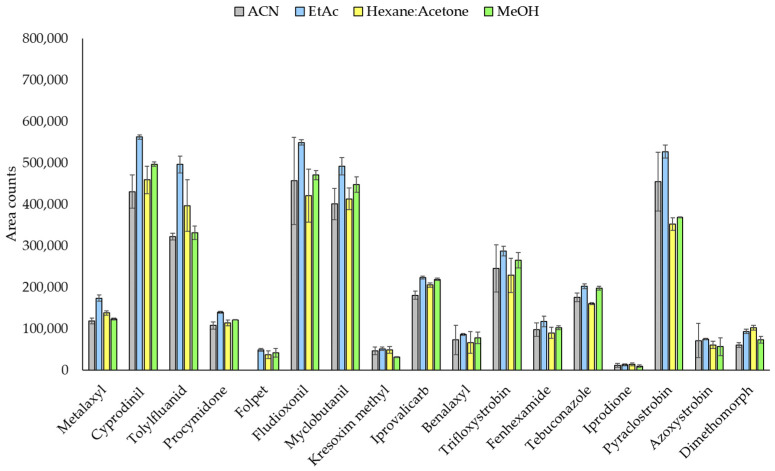
Fungicide peak areas for spiked vine leaves (400 ng g^−1^) extracted by UAE using different solvents: ACN, EtAc, Hexane:Acetone (50:50, *v*/*v*) and MeOH.

The solvent employed can be a crucial factor for the efficiency of the extraction process. In this way, UAE was carried out for 10 min using a spiked leaf sample (400 ng g^−1^) with different solvents: ACN, EtAc, Hexane:Acetone (50:50, *v*/*v*) and MeOH. The responses obtained are shown in Figure 2. As can be seen, the results were generally satisfactory and equivalent. A one-way ANOVA was performed to evaluate if statistically significant differences existed between the different solvents (Table S2). ANOVA demonstrated non-significant differences between the four solvents, excluding folpet with a *p*-value lower than 0.05 indicating statistical significance at the 95% confidence level. This result seemed to be due to the chromatographic analysis since this compound showed a very low or even no chromatographic response using ACN (see Section 3.1 and Figure S1). In addition, a multiple-range least significant difference (LSD) test was performed to pairwise compare the four solvents. As can be seen in Figure 2 and Table S2, some target compounds offered higher responses with EtAc with statistically significant differences: tolylfluanid (ACN-EtAc and EtAc-Hexane:Acetone), folpet (ACN-EtAc, ACN-Hexane:Acetone, ACN-MeOH and EtAc-Hexane:Acetone) and pyraclostrobin (EtAc-Hexane:Acetone and EtAc:MeOH). Kresoxim methyl (statistically significant for EtAc-MeOH and Hexane:Acetone-MeOH) and iprodione (statistically significant for Hexane:Acetone-MeOH) were less efficiently extracted using MeOH. However, azoxystrobin responses showed statistical differences with ACN-Hexane:Acetone, EtAc-Hexane:Acetone and EtAc:MeOH, with higher signals when ACN or EtAc were employed. Therefore, EtAc was chosen as the extraction solvent since higher responses were generally obtained for all fungicides.

**Figure 3:**
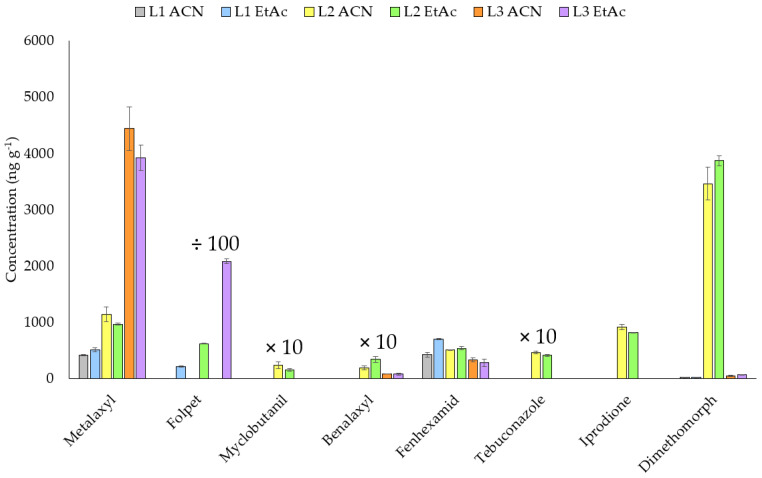
Comparison of ACN and EtAc in vine leaves which contain fungicides. ÷100: concentration of folpet divided by 100; ×10: concentration of myclobutanil, benalaxyl and tebuconazole multiplied by 10.

The efficiency of the extraction using EtAc and ACN was also compared in three leaf samples collected from treated vineyards. As shown in Figure 3, the results were equivalent for the compounds found in the samples, excluding folpet for which no response was obtained using ACN. Thus, EtAc was the solvent finally selected as the most favorable.

#### 3.2.2. Amount of Sample and Solvent and Multiple Extraction

The amount of sample (0.15 to 0.5 g) was tested maintaining the same solvent dilution (1:10 *w*/*v*) and simultaneous extraction was also evaluated (up to 6 samples). The results obtained were equivalent in all cases (data not shown), since no significant differences were found regardless of the relative amounts of leaf sample and solvent. Therefore, the final selected conditions included the use of UAE with 0.2 g of sample (leaf) and 2 mL of EtAc for 10 min. During this time, multiple extraction can be performed.

#### 3.2.3. Fresh and Dried Leaves

**Figure 4:**
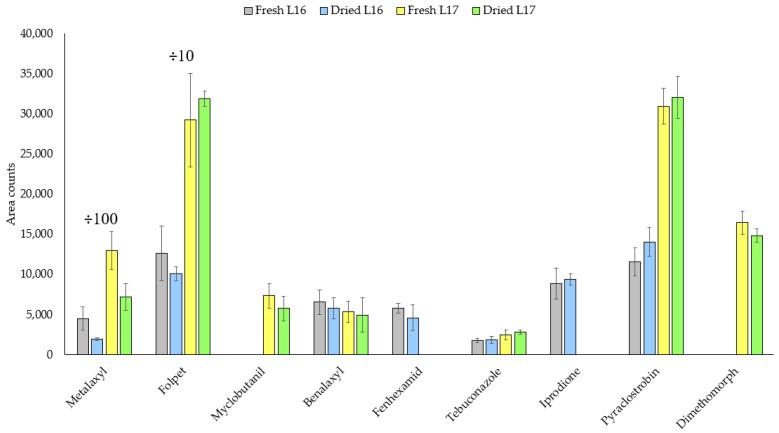
Fungicide responses for dried and fresh leaves in two real samples (L16 and L17). ÷100: area counts of metalaxyl divided by 100; ÷10: area counts of folpet divided by 10.

The effect of desiccation of the vine leaves on the extraction yield was evaluated since they contained high moisture at the time of their collection. For this study, two leaves treated with fungicides were dried at 80 °C for 3 h before performing the UAE method and the results were compared with those obtained with the fresh samples. As shown in Figure 4, both protocols can be applied for the analysis of the target fungicides, except for metalaxyl whose signal is reduced by drying the leaves. Nevertheless, it appears to be an interesting approach since the drying of the leaves is a common step to preserve the samples avoiding their deterioration [14]. In addition, some commercial products based on leaves, such as tea, are sold in dry format (small pieces or powder).

#### 3.2.4. Method Performance in Real Samples

Real vine leaves were fortified at three concentration levels: 40, 400 and 4000 ng g^−1^, aiming at evaluating method accuracy since reference materials were not available. UAE-GC-MS/MS analysis was performed, and recoveries were evaluated in triplicate by external calibration as the ratio of concentrations between found/added considering the responses obtained for each analyte. The results are included in Table 2. Quantitative results were obtained for all compounds with recovery values between 80 and 113% in almost all cases and at the three concentration levels. Precision was also satisfactory with RSD values below 9% for the medium and the higher level, and about 10% for the low concentration level.

**Figure 5:**
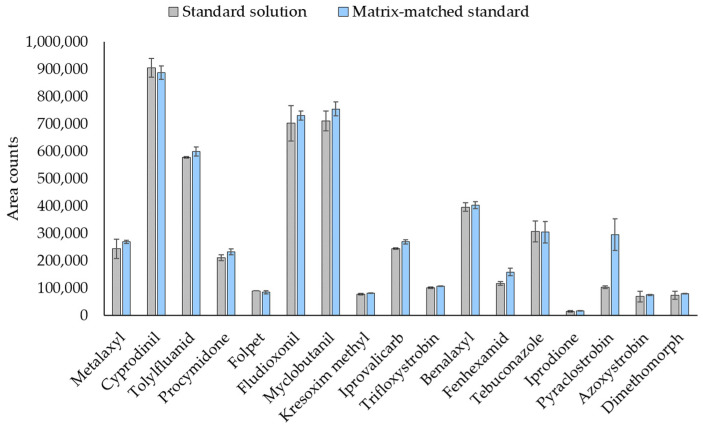
Fungicide responses for a standard solution and a matrix-matched standard at 10 µg L^−1^.

However, too high recovery values were achieved for fenhexamid and, especially, for pyraclostrobin. The matrix effect was then evaluated for these two compounds by comparing the slopes obtained in solvent calibration and in matrix-matched calibration, in the same concentration range (from 1 to 100 µg L^−1^) (data not shown). The ratios between the two slopes (matrix/solvent) were 1.3 and 2.7 for fenhexamid and pyraclostrobin, respectively. As an example, Figure 5 shows the comparison between a standard solution in solvent with a matrix-matched solution. As can be seen, signals at 10 µg L^−1^ were similar for all fungicides except for fenhexamid and pyraclostrobin, which exhibited higher responses with matrix-matched standards.

Therefore, quantification can be performed by external calibration, employing standards prepared in EtAc, for all studied fungicides, excluding fenhexamid and pyraclostrobin. For these compounds, matrix-matched calibration is highly recommended (estimated recovery 87–106% for the three concentration levels).

Method suitability was also evaluated in different plant materials such as tea leaves. For this study, commercial samples were acquired in local markets and a pool composite sample was prepared. The sample was spiked with all target compounds. Recoveries were also satisfactory with general values above 80% (Table 2). In this case, quantitative recovery values were also obtained for fenhexamid and pyraclostrobin, as there was no significant matrix effect.

To demonstrate the method suitability for other types of leaf samples, 8 tea samples were also analyzed in triplicate. Metalaxyl was detected in all samples in a concentration range of 2.0–7.5 ng g^−1^. Concentrations for each sample are summarized in Table S3. Cyprodinil was found only in one sample (Tea 3, a bulk sample) at 8 ng g^−1^. According to these results, the concentrations of the two detected fungicides did not exceed the MRLs in teas (50 and 100 ng g^−1^).

#### 3.2.5. Analysis of Real Samples

**Figure 6:**
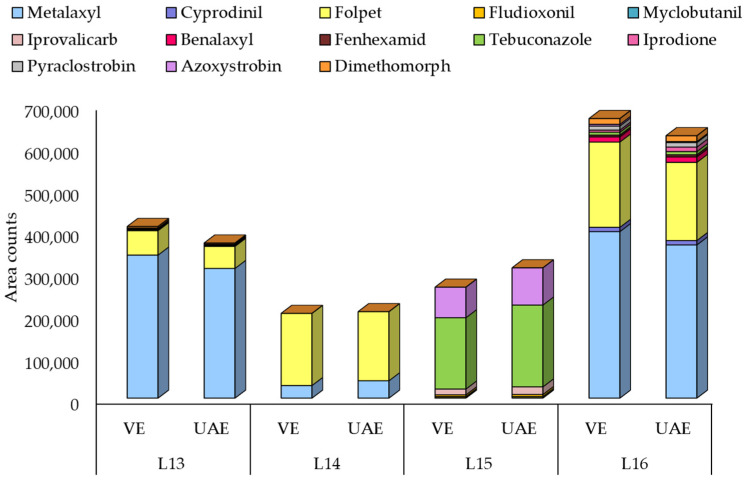
Comparison of the response of fungicides in four contaminated vine leaf samples extracted with UAE and VE. To visualize all the samples in the same scale, responses for L13 and L15 were divided by 10 and 1000, respectively.

Real contaminated samples of vine leaves from different origins were collected in Galicia (NW Spain). Extractions were performed in triplicate using the UAE method. In addition, VE was employed for some of the samples and the results were compared. Results for four real contaminated samples are depicted in Figure 6 (accumulative response of the target pesticides) and Table S4 (individual concentrations and RSD), demonstrating similar responses regardless of the extraction technique employed. Therefore, both extraction techniques proved suitable for the extraction of fungicides in vine leaves.

The concentration of fungicides in 17 samples analyzed are shown in Table 3. The presence of 14 out of the 17 target compounds was demonstrated in the analyzed samples. Metalaxyl and folpet were found in 14 of the 17 samples, followed by tebuconazole in 9, dimethomorph in 8, fenhexamid in 6 and iprovalicarb and azoxystrobin in 5. Benalaxyl, myclobutanil and pyraclostrobin were detected in 4 and 3 samples, respectively, and kresoxim methyl and iprodione in 2 samples. Finally, cyprodinil and fludioxonil were identified only in one sample (L15). The highest number of fungicides per sample was detected in L2 and L16, with the presence of 8 of the 17 studied compounds. The other samples contained between 2 and 7 fungicides, while one of the samples (L6) only contained one target fungicide (kresoxim methyl). It is worth noting the high concentrations of folpet in all samples, covering a range between 1 and 1078 μg g^−1^, which is in concordance with those obtained in other studies [29]. However, sample L16 showed the highest concentration of some target fungicides (cyprodinil, fludioxonil, iprovalicarb, tebuconazole and azoxystrobin).

The method was applied to 18 samples, from vines on which fungicides were applied with a drone in the frame of an experimental study. The leaves of the plants were treated with azoxystrobin and tebuconazole. Leaves were collected at three heights to assess the active substance dispersion over the entire vine (see samples L9–L12 and L15 given as an example in Table 3). According to the individual and mean concentrations of azoxystrobin and tebuconazole in the 18 samples (data not shown), it could be concluded that there were some differences in the concentrations of tebuconazole and azoxystrobin between the three heights/strata. As expected, the higher concentrations of both fungicides were observed in the upper stratum, but taking that level as a reference, we observed that 86% and 81% of the amounts of tebuconazole and azoxystrobin, respectively, remained at the lower stratum.

Metalaxyl, cyprodinil, folpet, iprovalicarb, fenhexamid, pyraclostrobin and dimethomorph exceeded MRLs for vine leaves in all samples in which they were detected.

As expected, since samples were collected just after fungicide applications by means of a drone, tebuconazole and azoxystrobin also exceeded these MRLs in the five samples belonging to the experimental study. Actually, L15 was treated with the two fungicides and the final measured concentrations were higher than 400 μg g^−1^ for both active substances.

The high presence of fungicides in this type of sample poses a problem for the environment, as residues can reach the soil via rainwater and irrigation water. Furthermore, these compounds can enter aquatic ecosystems through runoff water [2,8].

In order to demonstrate the method suitability for other types of leaf samples, 8 tea samples were also analyzed in triplicate. Metalaxyl was detected in all samples in a concentration range of 2.0–7.5 ng g^−1^. Fungicide concentrations for each sample are summarized in Table S4. Cyprodinil was found only in one sample (Tea 3, a bulk sample) at 8 ng g^−1^. According to these results, the concentrations of the two detected fungicides did not exceed the MRLs in teas (50 and 100 ng g^−1^).

### 3.3. Grapes

Seven grape samples collected in the vineyards were also extracted in triplicate by UAE. The concentration of the detected fungicides is shown in Table 4. Seven of the 17 target fungicides were detected in grape samples. Metalaxyl was found in all samples, folpet in 4, tebuconazole and dimethomorph in 3, benalaxyl in 2 and tolylfluanid and fludioxonil in only one sample. Tolylfluanid was the compound that reached the highest concentration, at 12,003 ng g^−1^ in sample G4. The highest concentration for metalaxyl, at 1833 ng g^−1^, was also measured in the same sample. In both cases, in sample G4, concentrations were higher than MRLs in grapes. For the other samples, only metalaxyl in sample G5 also exceeded the MRLs in wine grapes.

Samples G5, G6 and G7 correspond to the crop of the leaves L13, L16 and L17, respectively. Results are in concordance since fungicides that were present in the leaves were also detected in each grape sample. Concentration levels were lower in grapes, probably because the leaves protect them at the time of spraying and the surface wash-off effects were stronger for grapes than for leaves [30].

### 3.4. Soils

**Figure 7:**
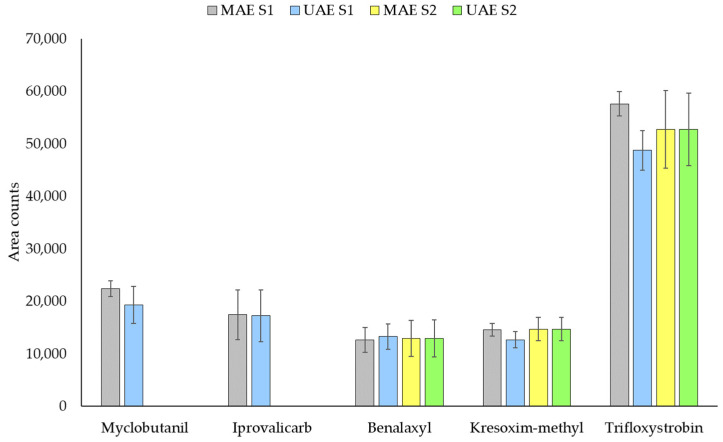
Comparison of UAE and MAE in soil samples (S1 and S2).

With the aim of studying the possible transfer of the fungicides into the soil, some soil samples were collected in the vineyards. For their analysis, the microwave extraction USEPA method 3546 [28] was adapted (see Section 2.3) and compared with UAE applying the same conditions as for the vine leaves. Results for some real samples are shown in Figure 7. As can be seen, some of the target compounds were found in the samples and the results obtained with both methodologies were in general comparable. Thus, the UAE method was selected considering its great ease of implementation as well as the need to use less solvent.

The results obtained for the samples analyzed in triplicate are shown in Table 5. Six of the 17 target analytes were found in the 4 analyzed samples. Benalaxyl, kresoxim methyl and trifloxystrobin were present in all samples. In addition, iprodione reached the highest concentration (15 μg g^−1^), but it was only detected in sample S4. Trifloxystrobin, which was not detected in any of the vine leaves, was present in all samples. In contrast, folpet, the compound detected in the largest number of leaf samples and at the highest concentrations, was not detected in soil. This could be due to the low to very low folpet persistence in soils, with DT50 usually below 4 days under field conditions, demonstrating its quick degradation in soil [31,32,33].

### 3.5. Waters

Water samples collected in the vineyards including rain and irrigation water were also analyzed in triplicate using a previously proposed methodology based on miniaturized SPE [8]. Of the target compounds, 14 out of the 17 were found in the 8 samples analyzed (Table 6). The 4 most frequently found compounds, like in the vine leaves, were metalaxyl and dimethomorph in 7 samples, and folpet and tebuconazole in 6 samples. It is important to highlight that two samples of rainwater, RW1 and RW2, were collected in the crop vineyards where leaf samples L13 and L15 were taken, respectively. The irrigation water samples IW1, IW2 and IW3 were also from the same places as leaf samples L14, L16 and L17, respectively. Sample RW2 reached the highest concentrations, up to 106 μg L^−1^, as did its corresponding leaf sample. These results demonstrate the diffusion and transport of fungicides applied on crops to water. Besides, the detected fungicides would exceed in all samples the EU maximum permitted concentration for pesticides in groundwater intended for human consumption (0.1 µg L^−1^ for individual pesticide and 0.5 µg L^−1^ for the sum of them) [11].

## 4. Conclusions

A method based on UAE, using EtAc as the solvent, followed by GC-MS/MS was developed for the simultaneous analysis of 17 fungicides in vine leaves. The suitability of the method was demonstrated for fresh and dried vine leaves as well as for other crops (tea). The methodology based on UAE-GC-MS/MS was successfully validated, showing quantitative recoveries. Finally, 17 vine leaf samples were analyzed revealing the presence of 14 of the 17 fungicides at concentrations up to 1000 μg g^−1^.

Grape, soil and water samples were also analyzed. Although MAE and UAE were suitable to extract analytes in soil samples, UAE was selected since it allowed performing the extraction with a minimal amount of solvent (2 mL). The concentration levels of the detected fungicides in soils ranged between 0.03 and 15 μg g^−1^. Grapes contained 7 of the 17 target fungicides and the similarity between the concentration profiles in grapes and in vine leaves was demonstrated. In addition, the 14 fungicides detected in water samples at concentrations up to 100 μg L^−1^ revealed pesticide transport from leaves to water. Therefore, the fungicide application modes should be improved since their residues can easily reach the surrounding environmental areas.

## Figures and Tables

**Table 1:** Target fungicides, CAS number, retention time, linearity, precision, IDLs and IQLs.

Fungicides	CAS	RT (min)	Linearity		Precision (RSD, %) ^b^		IDLs (μg L^−1^)	IQLs (μg L^−1^)
			Range (μg L^−1^)	R^2^	Intra-Day (*n* = 4)	Inter-Day (*n* = 6)		
Metalaxyl	57837-19-1	10.69	0.05–1000	0.9993	5.9	4.5	0.0012	0.0038
Cyprodinil	121552-61-2	11.61	0.2–1000	0.9989	7.7	5.8	0.042	0.14
Tolylfluanid	731-27-1	11.74	0.2–1000	0.9995	1.9	4.8	0.045	0.15
Procymidone	32809-16-8	11.88	0.05–1000	0.9999	11	11	0.0033	0.011
Folpet	133-07-3	12.01	10–1000	0.9946	6.4	15	1.5	5.1
Fludioxonil	131341-86-1	12.41	0.05–1000	0.9989	4.6	5.7	0.020	0.065
Myclobutanil	88671-89-0	12.64	0.02–1000	0.9993	4.8	15	0.0040	0.013
Kresoxim methyl	143390-89-0	12.65	0.5–1000	0.9988	8.2	7.7	0.045	0.15
Iprovalicarb	140923-17-7	12.69	0.2–1000	0.9937	7.1	6.9	0.041	0.14
Trifoxystrobin	141517-21-7	13.61	0.05–1000	0.9988	3.7	3.8	0.011	0.035
Benalaxyl	71626-11-4	13.65	0.02–1000	0.9990	2.6	3.7	0.0036	0.012
Fenhexamid	126833-17-8	13.97	0.5–1000	0.9941	12	8.1	0.080	0.26
Tebuconazole	80443-41-0	14.20	0.1–1000	0.9937	5.0	13	0.022	0.074
Iprodione	36734-19-7	14.67	2–1000	0.9980	6.2	7.0	0.36	1.2
Pyraclostrobin	175013-18-0	19.80	0.5–1000	0.9968	14	15	0.25	0.84
Azoxystrobin	131860-33-8	21.26	0.1–1000	0.9946	5.5	14	0.019	0.062
Dimethomorph ^a^	113210-97-2	21.50/22.14	0.1–1000	0.9966	7.8	5.9	0.019	0.061

^a^ This compound is a mixture of isomers. ^b^ 10 μg L^−1^, similar values were obtained for other concentration levels.

**Table 2:** Fungicide recoveries obtained for vine and tea leaves.

Recovery (%) (*n* = 3)	Vine Leaves				Tea Leaves
	40 ng g^−1^	400 ng g^−1^	4000 ng g^−1^	Mean	400 ng g^−1^
Metalaxyl	94 ± 9	110 ± 5	93 ± 3	99 ± 9	106 ± 16
Cyprodinil	83 ± 8	83 ± 0.8	94 ± 2	87 ± 6	93 ± 2
Tolylfluanid	91 ± 8	98 ± 4	97 ±1	95 ± 4	83 ± 3
Procymidone	88 ± 14	79 ± 2	91 ± 2	86 ± 6	96 ± 0.5
Folpet	-	63 ± 8	97 ± 16	80 ± 23	81 ± 9
Fludioxonil	100 ± 11	113 ± 1	105 ± 5	106 ± 6	95 ± 1
Myclobutanil	111 ± 7	97 ± 4	91 ± 5	99 ± 10	92 ± 1
Kresoxim methyl	102 ± 9	82 ± 7	100 ± 2	95 ± 11	92 ± 7
Iprovalicarb	106 ± 12	114 ± 2	93± 7	104 ± 11	93 ± 5
Trifloxystrobin	95 ± 12	109 ± 4	108 ± 2	104 ± 7.8	96 ± 1
Benalaxyl	103 ± 13	101 ± 4	93 ± 1	99 ± 5.2	97 ± 4
Fenhexamid *	137 ± 11	127 ± 7	135 ± 7	133 ± 5.5	105 ± 1
Tebuconazole	92 ± 8	118 ± 3	97 ± 5	102 ± 14	101 ± 11
Iprodione	-	101 ± 3	102 ± 9	101 ± 0.3	91 ± 2
Pyraclostrobin *	265 ± 11	291 ± 9	252 ± 4	270 ± 20	95 ± 4
Azoxystrobin	108 ± 10	111 ± 2	120 ± 4	113 ± 6.7	89 ± 1
Dimethomorph	109 ± 7	113 ± 6	116 ± 5	113 ± 3.4	69 ±2

* Corrected values for each corresponding level: fenhexamid: 106, 98 and 104%; pyraclostrobin: 92, 100 and 87%.

**Table 3:** Concentration ± standard deviation (SD) (µg g^−1^) of the fungicides in vine leaves analyzed by UAE-GC-MS/MS.

Samples (*n* = 3)	Metalaxyl	Cyprodinil	Folpet	Fludioxonil	Kresoxim Methyl	Mycobutanil	Iprovalicarb	Benalaxyl	Fenhexamid	Tebuconazole	Iprodione	Pyraclostrobin	Azoxystrobin	Dimethomorph
L1	0.51 ± 0.04		22 ± 1						0.70 ± 0.01					0.023 ± 0.002
L2	1.0 ± 0.2		62 ± 0.8			0.016 ± 0.002		0.034 ± 0.006	0.54 ± 0.03	0.041 ± 0.014	0.81 ± 0.01			3.9 ± 0.1
L3	3.9 ± 0.2		208 ± 43					0.0079 ± 0.0014	0.28 ± 0.07					0.069 ± 0.003
L4	11 ± 3		8.2 ± 1.0									0.25 ± 0.05		0.075 ± 0.009
L5	3.9 ± 0.2								0.12 ± 0.03					
L6					0.23 ± 0.01									
L7	1.9 ± 0.37				0.19 ± 0.04				0.12 ± 0.01					
L8	0.082 ± 0.023		35 ± 7											
L9	0.13 ± 0.05		277 ± 26				9.8 ± 2.4			4.2 ± 1.3			13 ± 0.79	
L10			15 ± 1				3.3 ± 0.7			2.0 ± 0.36			60 ± 6.5	
L11	0.18 ± 0.02		1078 ± 211				21 ± 4			30 ± 3			2.0 ± 0.3	
L12	0.061 ± 0.017		14 ± 3				1.3 ± 0.2			23 ± 1			0.098 ± 0.003	
L13	7.4 ± 0.3		3.3 ± 0.2			0.013 ± 0.004			0.058 ± 0.006	0.013 ± 0.002		0.035 ± 0.002		0.17 ± 0.01
L14	0.065 ± 0.004		1.0 ± 0.2											
L15		0.46 ± 0.13	18 ± 2	3.3 ± 0.8			39 ± 13			461 ± 50			661 ± 54	0.18 ± 0.01
L16	0.87 ± 0.16		1.2 ± 0.1					0.013 ± 0.001	0.022 ± 0.001	0.012 ± 0.001	0.30 ± 0.03	0.021 ± 0.001		0.064 ± 0.007
L17	5.0 ± 0.9		1.9 ± 0.4			0.0063 ± 0.0010		0.010 ± 0.002		0.0052 ± 0.0013		0.085 ± 0.006		0.060 ± 0.005
MRLs in vine leaves	0.01	0.02	0.03	0.01	15	0.05	0.01	0.05	0.01	0.02	0.01	0.02	0.01	0.01

**Table 4:** Concentration ± SD (ng g^−1^) of the target fungicides in grape samples by UAE-GC-MS/MS.

Samples (*n* = 3)	Metalaxyl	Tolylfluanid	Folpet	Fludioxonil	Benalaxyl	Tebuconazole	Dimethomorph
G1	194 ± 7		982 ± 36	2.6 ± 0.42			4.8 ± 0.7
G2	242 ± 15						
G3	150 ± 5						
G4	1833 ± 44	12,003 ± 453	535 ± 70				
G5	1436 ± 28		943 ± 61			4.1 ± 0.3	
G6	29 ± 1				16 ± 4	11 ± 2	58 ± 5
G7	515 ± 7		403 ± 51		1.2 ± 0.2	11 ± 3	3.3 ± 0.8
MRLs in grapes (Table/Wine)	2000/1000	20/20	6000/20,000	5000/4000	300/300	500/1000	3000/3000

**Table 5:** Concentration ± SD (ng g^−1^) of fungicides in soil samples by UAE-GC-MS/MS.

Samples (*n* = 3)	Myclobutanil	Iprovalicarb	Benalaxyl	Kresoxim Methyl	Trifloxystrobin	Iprodione
S1	51 ± 3	115 ± 4	27 ± 5	140 ± 17	526 ± 40	
S2			31 ± 2	189 ± 25	720 ± 100	
S3		113 ± 19	30 ± 6.8	550 ± 48	682 ± 21	15,337 ± 275
S4		167 ± 21	32 ± 2.1	276 ± 50	803 ± 24	

**Table 6:** Concentration ± SD (μg L^−1^) of detected fungicides in rainwater (RW) and irrigation water (IW).

Samples (*n* = 3)	Metalaxyl	Cyprodinil	Folpet	Fludioxonil	Myclobutanil	Kresoxim Methyl	Iprovalicarb	Benalaxyl	Fenhexamid	Tebuconazole	Iprodione	Pyraclostrobin	Azoxystrobin	Dimethomorph
RW1	7.0 ± 0.8		83 ± 2		0.075 ± 0.001					0.17 ± 0.02				0.86 ± 0.31
RW2		0.020 ± 0.002	73 ± 23	0.47 ± 0.02			0.46 ± 0.10			106 ± 2			41 ± 5.3	
RW3	14 ± 3				0.025 ± 0.010	0.14 ± 0.03		0.036 ± 0.013	0.052 ± 0.013	0.028 ± 0.003				0.33 ± 0.07
RW4	9.1 ± 1.2				0.010 ± 0.001	0.15 ± 0.02		0.020 ± 0.010	0.20 ± 0.02					0.26 ± 0.10
IW1	0.15 ± 0.03		12 ± 2											0.049 ± 0.017
IW2	0.72 ± 0.16		19 ± 1					0.016 ± 0.004	0.029 ± 0.014	0.036 ± 0.010	0.35 ± 0.013	0.076 ± 0.0044		0.13 ± 0.010
IW3	5.1 ± 0.9		41 ± 10					0.019 ± 0.003	0.40 ± 0.040	0.082 ± 0.031		0.13 ± 0.021		0.40 ± 0.11
IW4	8.6 ± 0.6		2.3 ± 0.5					0.069 ± 0.010	0.18 ± 0.04	0.022 ± 0.006				0.20 ± 0.042

## Data Availability

Data are available within the present article and Supplementary Materials.

## Supplementary Materials

The following are available online, Figure S1: Comparison of fungicide responses obtained in ACN, EtAc, Hexane:acetone (50:50, *v*/*v*) and MeOH for standard solutions at 100 μg L^−1^. Table S1: MS/MS transitions of target fungicides. Underlined MS/MS transition is the one selected for quantification. Table S2: ANOVA (values in bold denote statistical significance: *p*-value < 0.05) and multiple range LSD test for the four studied solvents (* denote statistical significance), Table S3: Concentration ± SD (ng g^−1^) of metalaxyl in tea samples. Table S4: Concentrations ± SD (µg g^−1^) of the fungicides in vine leaves extracted with UAE and VE.

## Sample Availability

Samples analyzed in this study are not available from the authors.
