# Supplementary material for: Occurrence of Fungicides in Vineyard and the Surrounding Environment
Source: Molecules. 2021 Oct 12;26(20):6152. doi: 10.3390/molecules26206152 (PMC8537801; doi:10.3390/molecules26206152)
Supplement: Supplementary file 1 [file molecules-26-06152-s001.zip › molecules-1394936-supplementary.pdf]

Figure S1. Comparison of fungicide responses obtained in ACN, EtAc, Hexane:acetone (50:50, v/v) and MeOH, for standards solutions at 100 µg L^{-1}

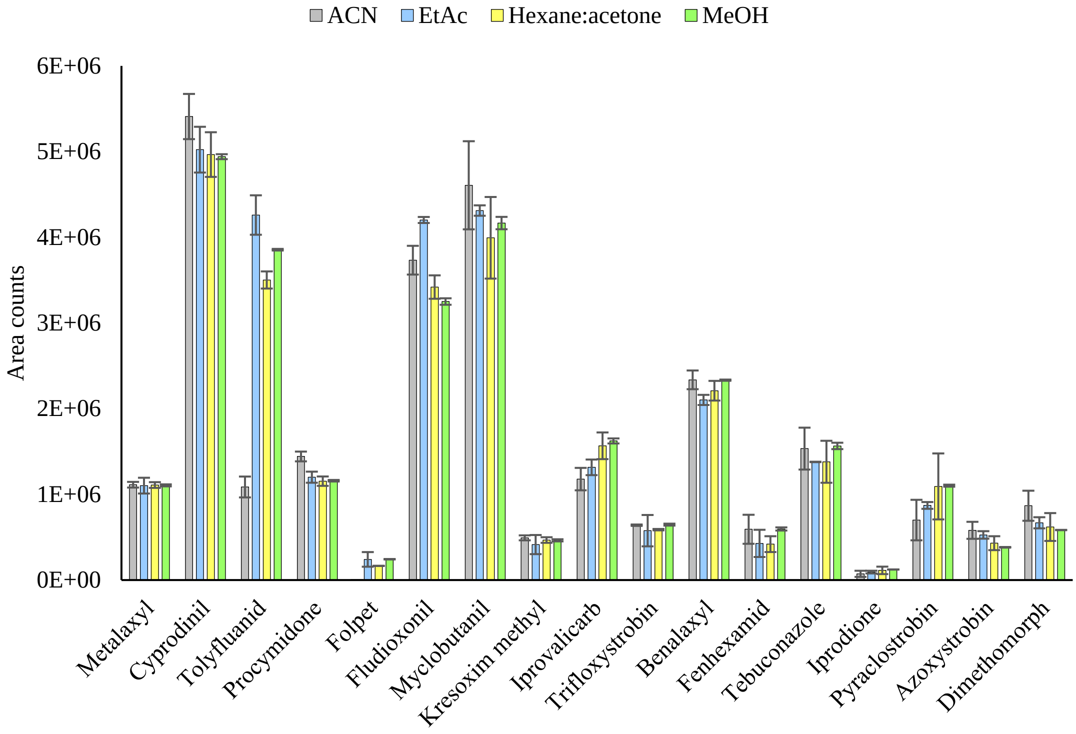

Table S1. MS/MS transitions of target fungicides. Underlined MS/MS transition is the selected for quantification.

**Fungicides**	**MS/MS transitions (Collision energy, eV)**
Metalaxyl	206.1 → 132.0 (15)
	234.1 → 174.1 (10)
Cyprodinil	224.1 → 208.1 (20)
	225.1 → 210.1 (18)
Tolylfluanid	137.0 → 91.0 (20)
	238.0 → 136.0 (15)
	238.0 → 137.0 (10)
Procymidone	283.0 → 96-0 (15)
	283.0 → 255.0 (10)
Folpet	146.9 → 102.9 (10)
	259.9 → 94.9 (20)
	259.9 → 129.9 (16)
Fludioxonil	248.0 → 127.0 (30)
	248.0 → 154 (20)
Myclobutanil	179.0 → 125.05 (15)
	179.0 → 152.0 (15)
Kresoxim methyl	206.1 → 116.0 (15)

	206.1 → 131.0 (15)
Iprovalicarb	116.1→ 55.1 (10)
	116.1 → 98.0 (10)
Trifoxystrobin	222.1 → 162.1 (10)
	222.1 → 190.1 (4)
Benalaxyl	234.1 → 174.1 (10)
	266.1 → 148.1 (10)
Fenhexamid	177.0 → 113.0 (10)
	178.9 → 113.1 (10)
	301.1 → 97.0 (15)
Tebuconazole	250.1 → 125.1 (20)
	252.1 → 127.1 (20)
Iprodione	187.0 → 124.0 (20)
	187.0 → 159.0 (40)
	314.0 → 245.0 (15)
Pyraclostrobin	132.0 → 77.0 (20)
	164.0 → 132.0 (15)
Azoxystrobin	344.1 → 156.0 (20)
	344.1 → 172.0 (20)
	388.1 → 345.1 (15)
Dimethomorph	301.1 → 165.0 (10)
	387.1 → 301.1 (12)

Table S2. ANOVA (values in bold denote statistical significance: p-value < 0.05) and multiple range LSD test for the four studied solvents (* denote statistical significance).

**Fungicides**	**F-ratio**	***P-value***	**ACN-EtAc**	**ACN-Hex:Acet.**	**ACN-MeOH**	**EtAc-Hex:Acet**	**EtAc-MeOH**	**Hex:Acet-MeOH**
Metalaxyl	2.87	0.1674						
Cyprodinil	1.99	0.2580						
Tolylfluanid	2.93	0.1630	*				*	
Procymidone	2.16	0.2352						
Folpet	**125.81**	**0.0002**	*	*	*	*		
Fludioxonil	1.71	0.3021						
Myclobutanil	1.17	0.4262						
Kresoxim methyl	5.07	0.0754					*	*
Iprovalicarb	1.76	0.2940						
Trifloxystrobin	1.62	0.3184						
Benalaxyl	1.24	0.4064						
Fenhexamid	1.73	0.2987						
Tebuconazole	1.69	0.3052						
Iprodione	5.16	0.0735						*
Pyraclostrobin	4.67	0.0854				*	*	
Azoxystrobin	5.59	0.0649		*		*	*	
Dimethomorph	0.41	0.7554						

Table S3. Concentration ± SD (ng g^{-1}) of Metalaxyl in tea samples.

**Samples (n=3)**	**Metalaxyl**
Tea 1	7.5 ± 0.4
Tea 2	2.1 ± 0.1
Tea 3	2.9 ± 0.3
Tea 4	3.2 ± 0.11
Tea 5	2.1 ± 0.3
Tea 6	6.6 ± 0.3
Tea 7	2.0 ± 0.3
Tea 8	2.3 ± 0.2

Table S4. Concentrations ± SD (μg g^{-1}) of the fungicides in vine leaves extracted with UAE and VE.

Samples (n=3)	Metalaxyl	Cyprodinil	Folpet	Fludioxonil	Mycobutanil	Iprovalicarb	Benalaxyl	Fenhexamid	Tebuconazole	Iprodione	Pyraclostrobin	Azoxystrobin	Dimethomorph
L13 VE	6.7 ± 0.3		3.0 ± 0.1		0.010 ± 0.002			0.045 ± 0.008	0.0090 ± 0.0009		0.037 ± 0.006		0.13 ± 0.01
L13 UAE	7.4 ± 0.3		3.3 ± 0.2		0.013 ± 0.004			0.058 ± 0.006	0.013 ± 0.002		0.035 ± 0.002		0.17 ± 0.01
L14 VE	0.090 ± 0.004		0.94 ± 0.10										
L14 UAE	0.065 ± 0.004		1.0 ± 0.2										
L14 VE		0.45 ± 0.13	17 ± 4	2.9 ± 0.8		48 ± 9			529 ± 79			787 ± 125	0.21 ± 0.08
L15 UAE		0.46 ± 0.13	18 ± 2	3.3 ± 0.8		39 ± 13			461 ± 50			661 ± 54	0.18 ± 0.01
L14 VE	0.80 ± 0.25		1.1 ± 0.1				0.014 ± 0.001	0.026 ± 0.007	0.013 ± 0.001	0.32 ± 0.02	0.019 ± 0.002		0.064 ± 0.004
L16 UAE	0.87 ± 0.16		1.2 ± 0.1				0.013 ± 0.001	0.022 ± 0.001	0.012 ± 0.001	0.30 ± 0.03	0.021 ± 0.001		0.064 ± 0.007
